# Supplementary material for: Yield reduction under climate warming varies among wheat cultivars in South Africa
Source: Nat Commun. 2020 Sep 2;11:4408. doi: 10.1038/s41467-020-18317-8 (PMC7468144; doi:10.1038/s41467-020-18317-8)
Supplement: Supplementary file 1 — Supplementary Information [file 41467_2020_18317_MOESM1_ESM.pdf]

SUPPLEMENTARY INFORMATION

Yield reduction under climate warming varies among wheat cultivars in South Africa

Shew et al.

SUPPLEMENTARY FIGURES

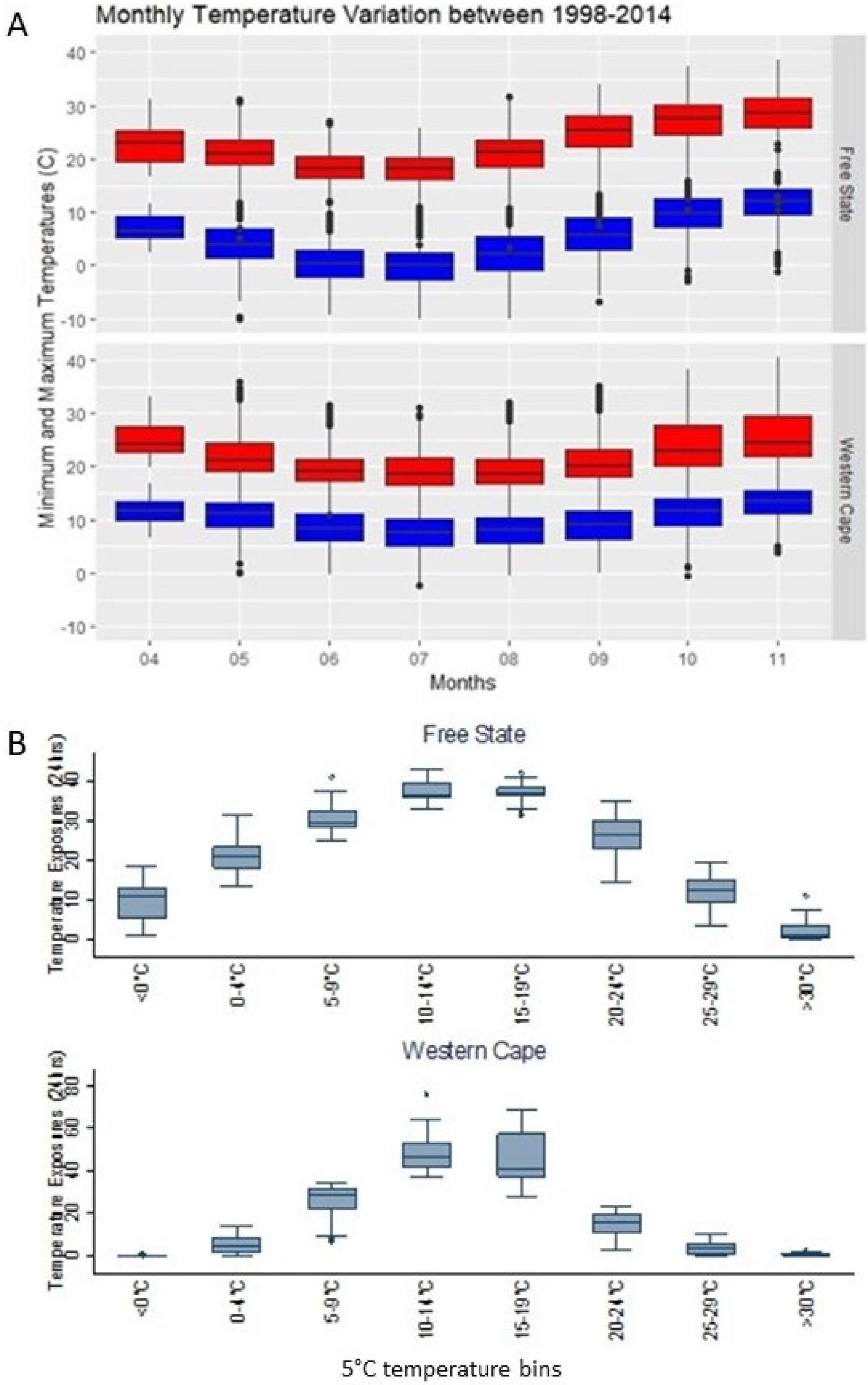

Supplementary Figure 1. Monthly Temperature Variation and Temperature Bin Exposures in Free State and Western Cape Provinces, 1998-2014. A. Minimum and maximum temperatures by month for Free State and Western Cape, respectively. Red boxes represent maximum temperatures while blue boxes represent minimum temperatures. Each box is defined by the upper and lower quartile for daily temperatures within the month, with the median depicted as a horizontal line within the box. The endpoints for the whiskers are the upper and lower adjacent values, which are defined as the relevant quartile +/- three-halves of the interquartile range, and circles represent data points outside of the adjacent values. **B.** Temperature bins of 5 degrees C are calculated as the summation of time (24 hour periods) spent within each temperature range and capture all temperatures within the 5 degree range up to the next bin for each of n=18,881 yield observations. This was based on a sinusoidal interpolation of daily minimum and maximum temperatures for the average growing season in each province. Each box is defined by the upper and lower quartile, with the median depicted as a horizontal line within the box. The endpoints for the whiskers are the upper and lower adjacent values, which are defined as the relevant quartile +/- three-halves of the interquartile range, and circles represent data points outside of the adjacent values. Exposures greater than 30°C occur substantially more in Free State compared to the Western Cape. Free State and Western Cape provinces account for 73% and 99% of total and dryland wheat production, respectively, in South Africa.

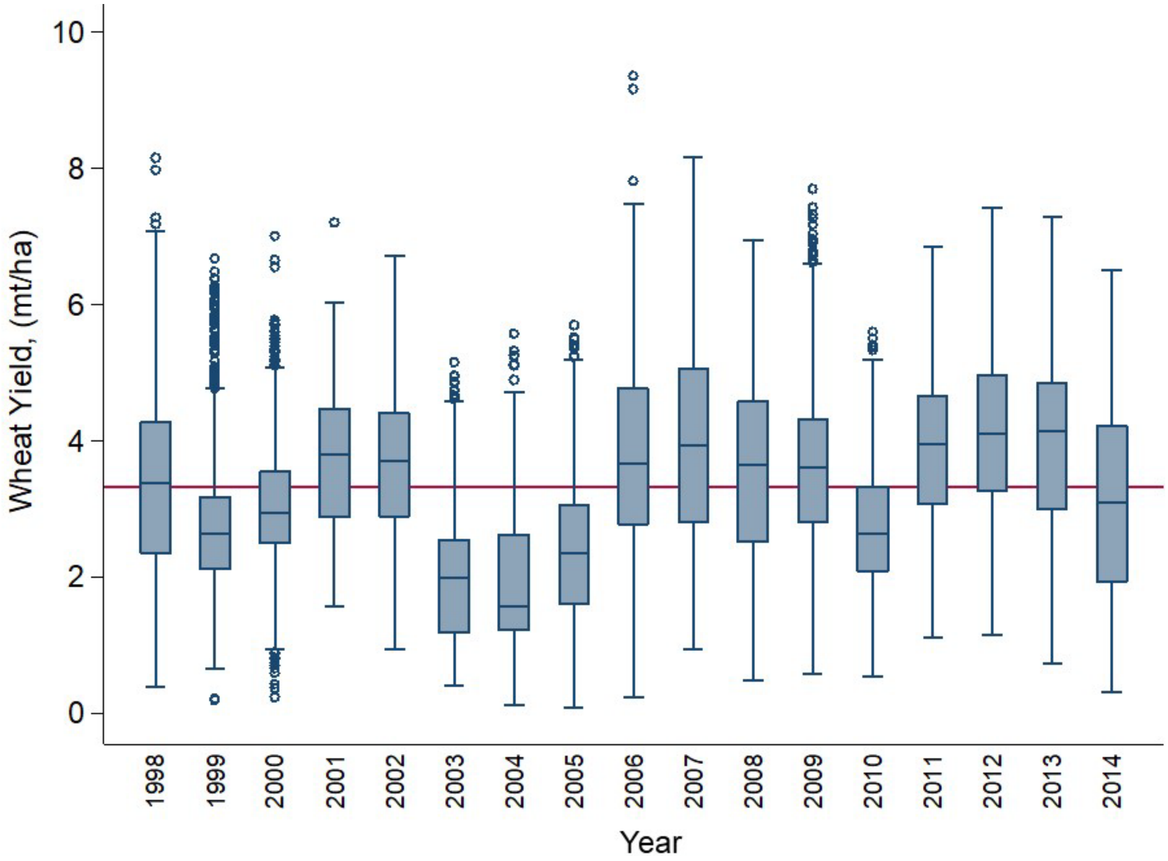

Supplementary Figure 2. Wheat yield (mt/ha) variability by year. We observe n=18,881 wheat yields at the trial location-year level and construct boxplots for each year from 1998 – 2014. Yields vary substantially from year to year. Each box is defined by the upper and lower quartile, with the median depicted as a horizontal line within the box. The endpoints for the whiskers are the upper and lower adjacent values, which are defined as the relevant quartile +/- three-halves of the interquartile range, and circles represent data points outside of the adjacent values.

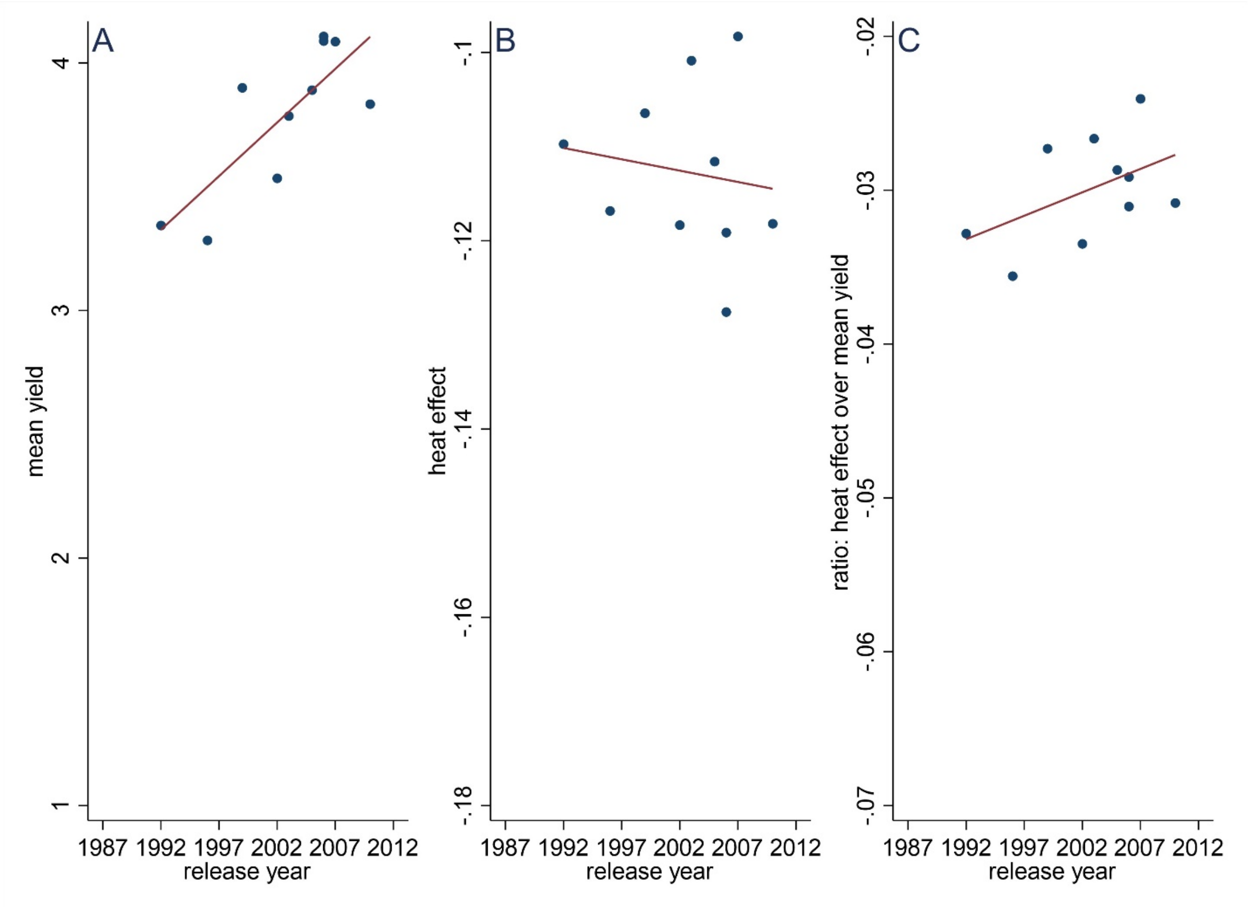

Supplementary Figure 3. Mean Yields and the Effect of Heat across Wheat Cultivar Release Years for the 10 Cultivars with the Highest Heat Ratios. The figure shows the tradeoff between mean yields and the effect of temperature occurrences above 30°C (heat effect) across cultivars based on the year they were publicly released. Data points are for specific cultivars with the 10 highest heat ratios from Figure 5 in the manuscript and lines are linear trends. Panels A, B, and C report values for mean yields, heat effects, and the heat effect normalized by mean yield, respectively, for the following cultivars: PAN3144, PAN3118, SST399, PAN3120, SST347, SST387, MATLABAS, TUGELA-DN, PAN3191, SST966.

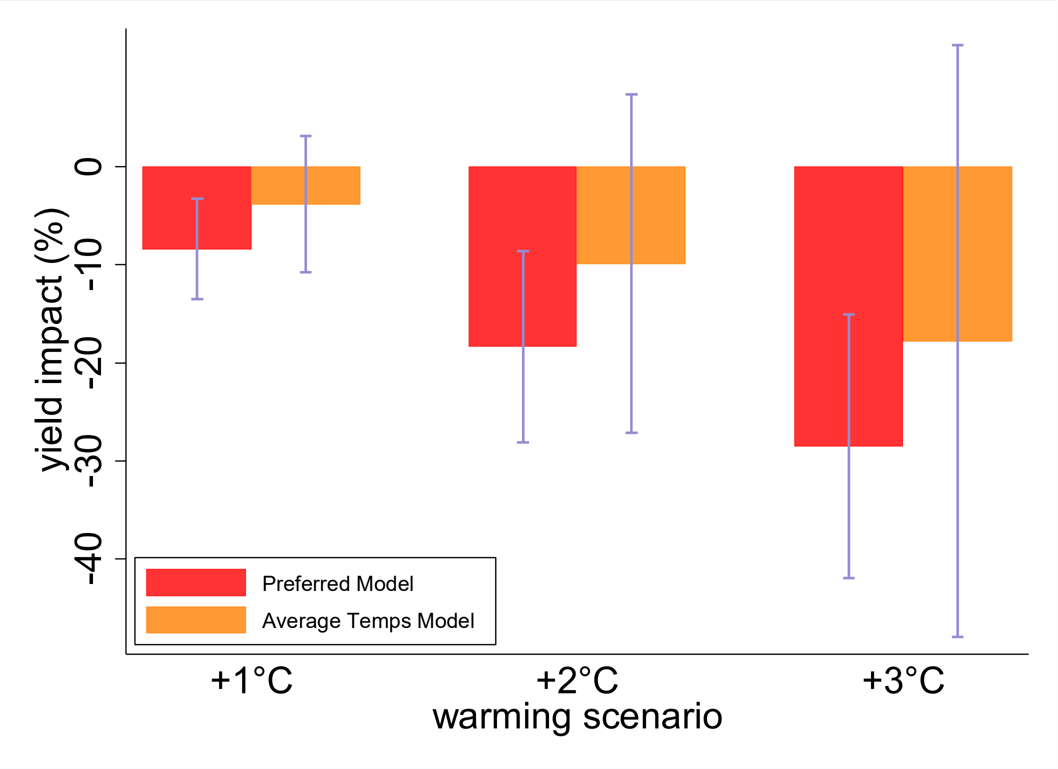

Supplementary Figure 4. Comparison of the Preferred Model of Temperature Exposure Bins with a Quadratic Function of Average Temperatures. Impacts are reported as the percentage change in mean yield relative to historical climate. The graph displays the warming effects for climate change scenarios based on our preferred model using temperature bins with warming scenarios for 1 to 3°C. Bars show 95% confidence intervals using standard errors clustered by province-year for n=18,881 yield observations. Each 2-bar cluster shows estimates for a given warming scenario across the two regression specifications, exposure bins versus average temperatures.

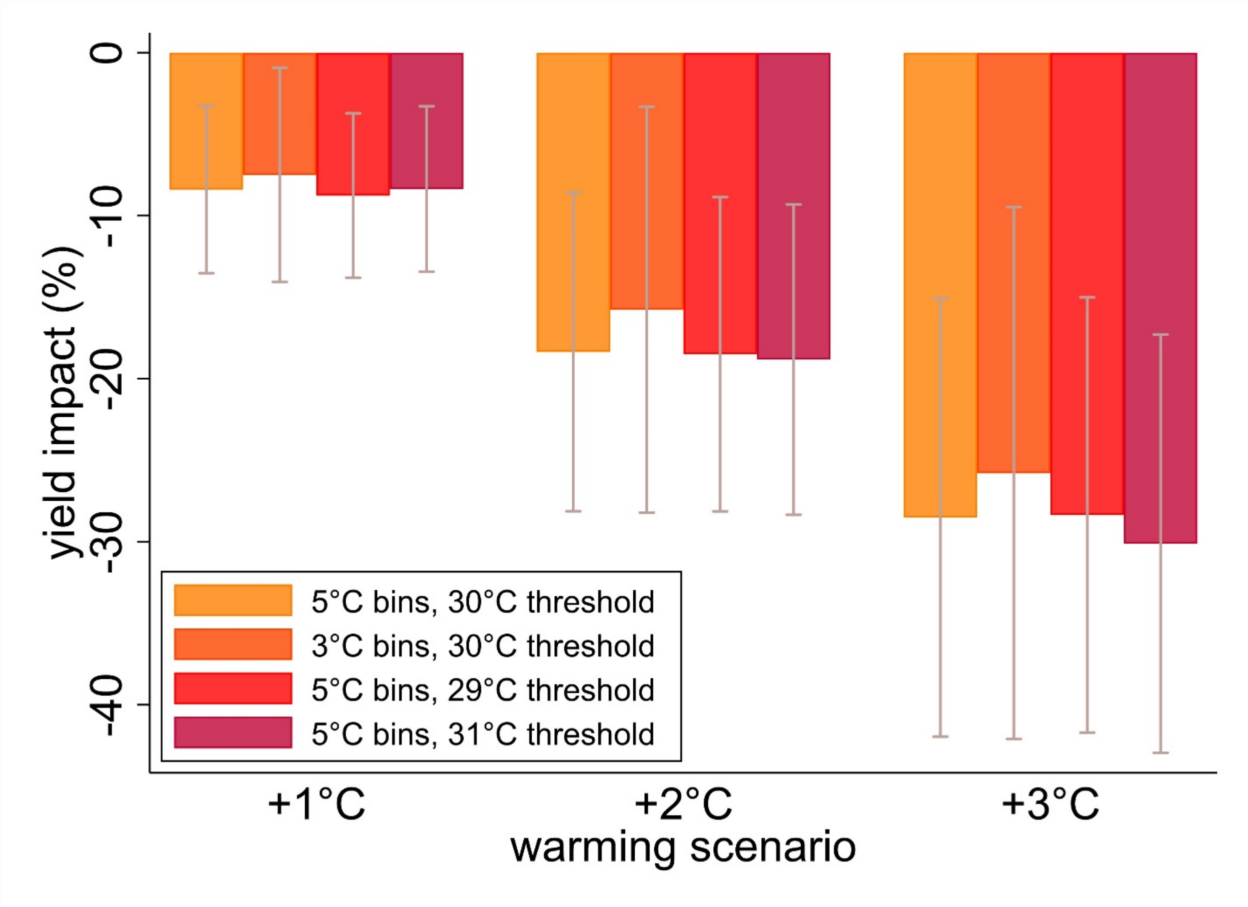

Supplementary Figure 5. Comparison of warming scenarios for four temperature bin and threshold selection models. The 5°C model is presented as the preferred model. For the 3°C model, the bins above zero are aggregated in subsequent temperature bins beginning with 0-2°C, 3-5°C, and so forth through 27-29°C, and then all temps above 30°C are aggregated into one upper bin. For the alternative upper temperature thresholds of 29°C and 31°C, respectively, we use the same bins as the initial 5°C model up to 24C, i.e., 0-4°C, 5-9°C, and so forth through 20-24°C. The upper bins are defined as 25-28°C and then 29°C + and as 25-30°C and then 31°C + for the new 29°C and 31°C threshold models, respectively. Within each warming scenario, we find similar marginal effects for both reduced bin size (3°C instead of 5°C) and two different upper thresholds. Bars show 95% confidence intervals using standard errors clustered by province-year.

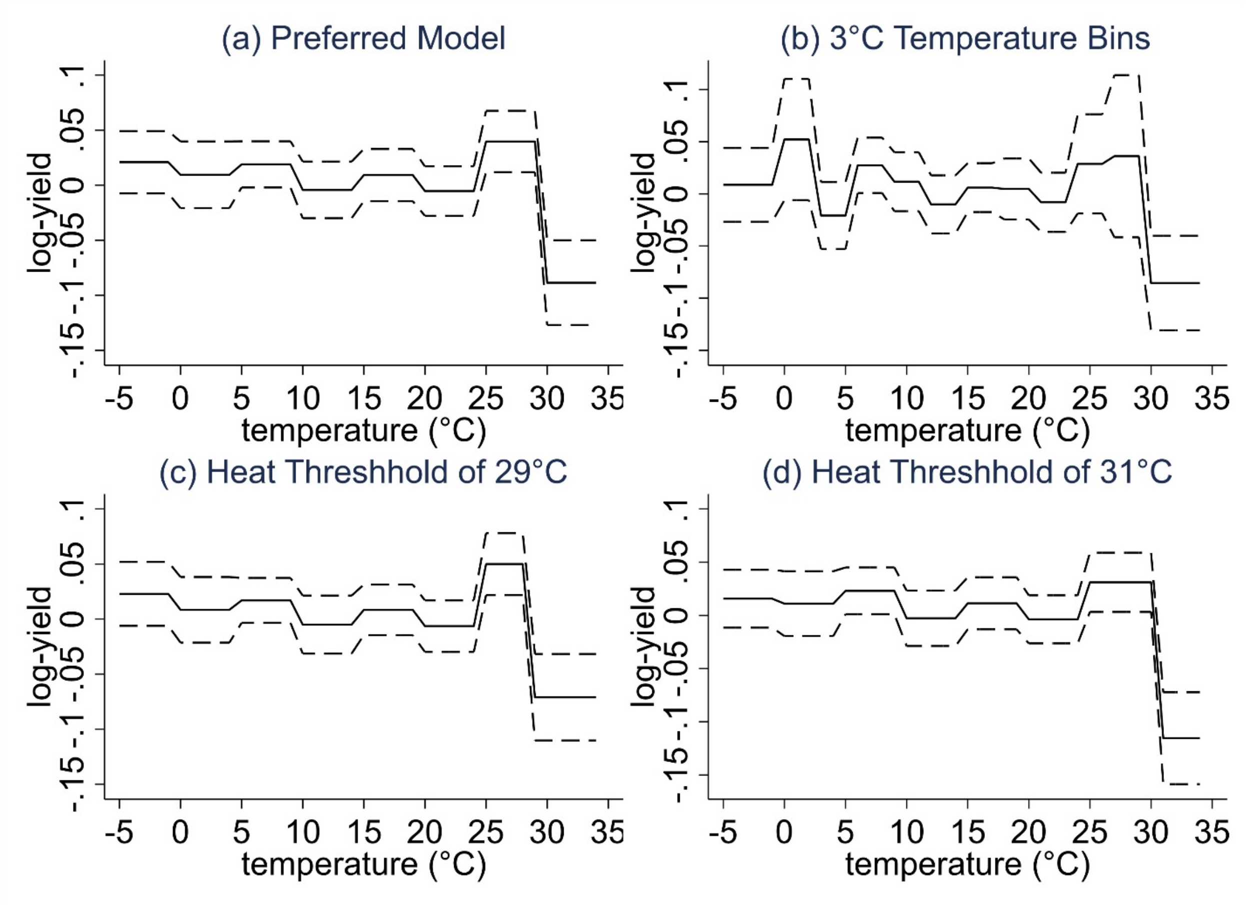

Supplementary Figure 6. Marginal Effects of Heat on Log Wheat Yield with Differing Temperature Bin Size and Upper Threshold. The 5°C model is presented as the preferred model. For the 3°C model, the bins above zero are aggregated in subsequent temperature bins beginning with 0-2°C, 3-5°C, and so forth through 27-29°C, and then all temps above 30°C are aggregated into one upper bin. For the alternative upper temperature thresholds of 29°C and 31°C, respectively, we use the same bins as the initial 5°C model up to 24C, i.e., 0-4°C, 5-9°C, and so forth through 20-24°C. The upper bins are defined as 25-28°C and then 29°C + and as 25-30°C and then 31°C + for the new 29°C and 31°C threshold models, respectively. Within each warming scenario, we find similar marginal effects for both reduced bin size (3°C instead of 5°C) and two different upper thresholds. Dashed lines represent the 95% confidence interval.

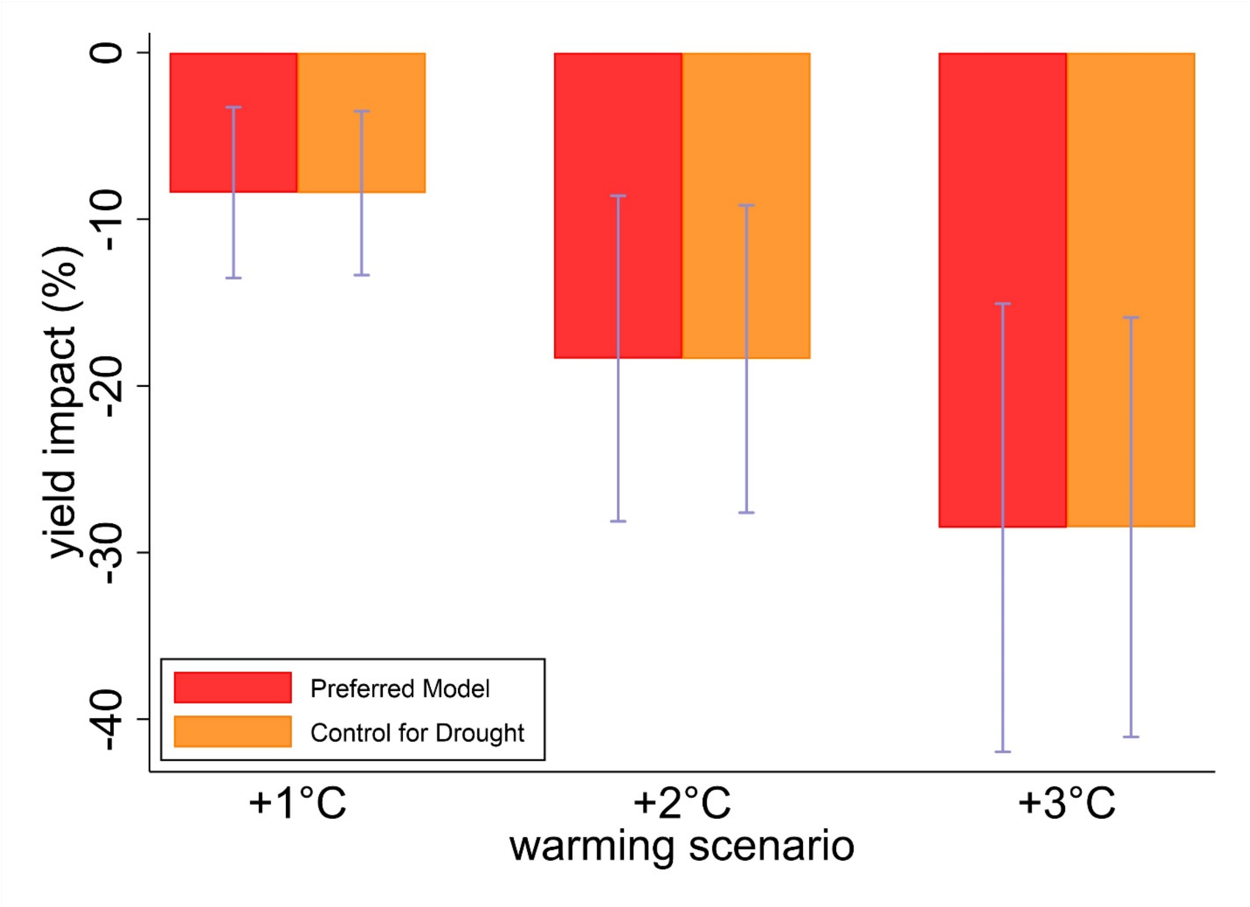

Supplementary Figure 7. Comparing Preferred Model for Warming Impacts with a Drought Control Variable. The control for drought model is specified similarly to preferred model with the exception that an interaction for 10th percentile rainfall is included. The figure above shows that the warming impacts for the preferred model and control for drought model are statistically similar, which suggests that drought impacts are differentiated from the heat impacts. Bars show 95% confidence intervals using standard errors clustered by province-year for n=18,881 yield observations.

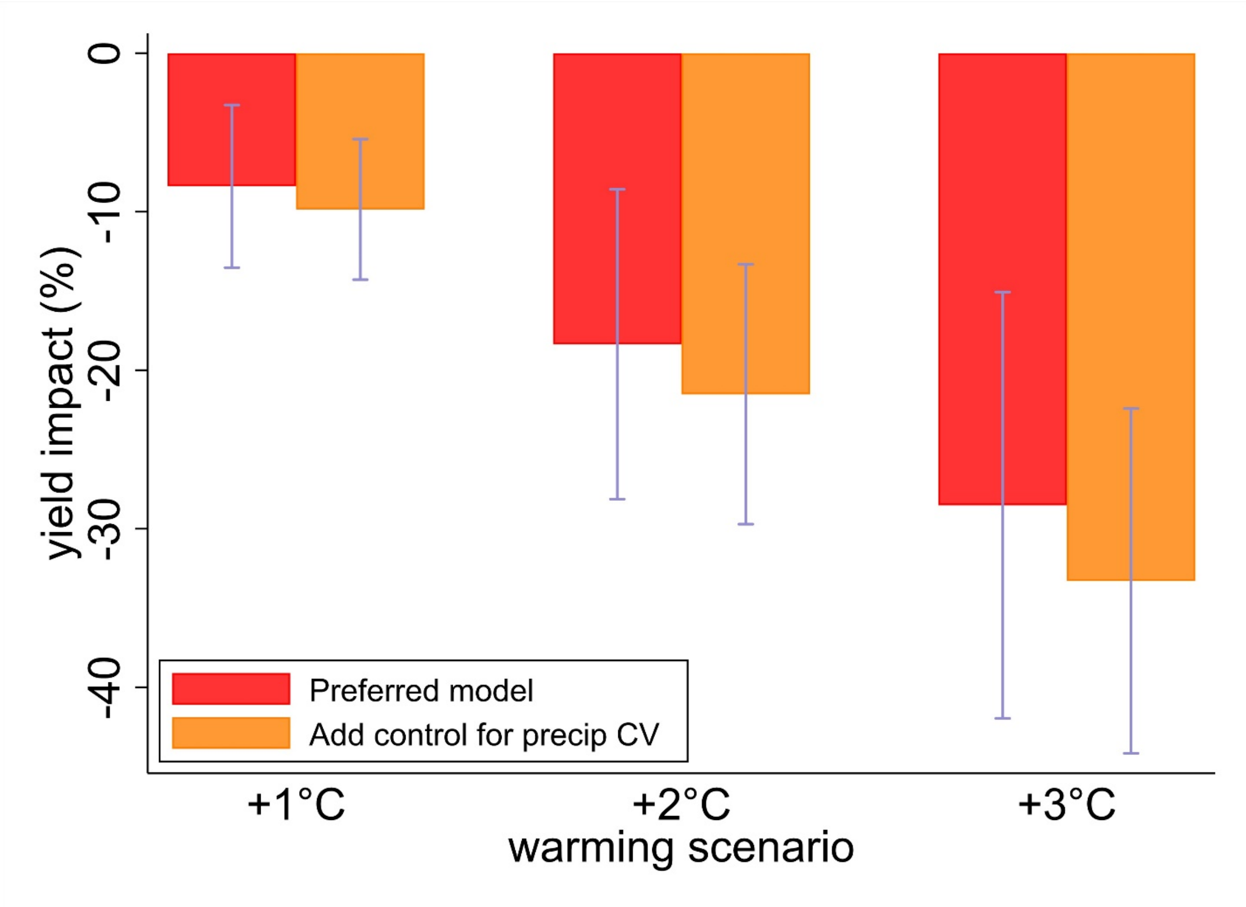

Supplementary Figure 8. Comparing Preferred Model for Warming Impacts with a Control for the Coefficient of Variation (CV) for Precipitation. After controlling for the seasonal variation in precipitation via the CV, the warming impacts for each scenario remain similar to the preferred model. This suggests that the high temperature effect and the precipitation effect are well-differentiated from each other, likely due to the location and year fixed effects that control for (among other things) locations with a more drought-prone climate and widespread droughts across locations within years. Bars show 95% confidence intervals using standard errors clustered by province-year for n=18,881 yield observations.

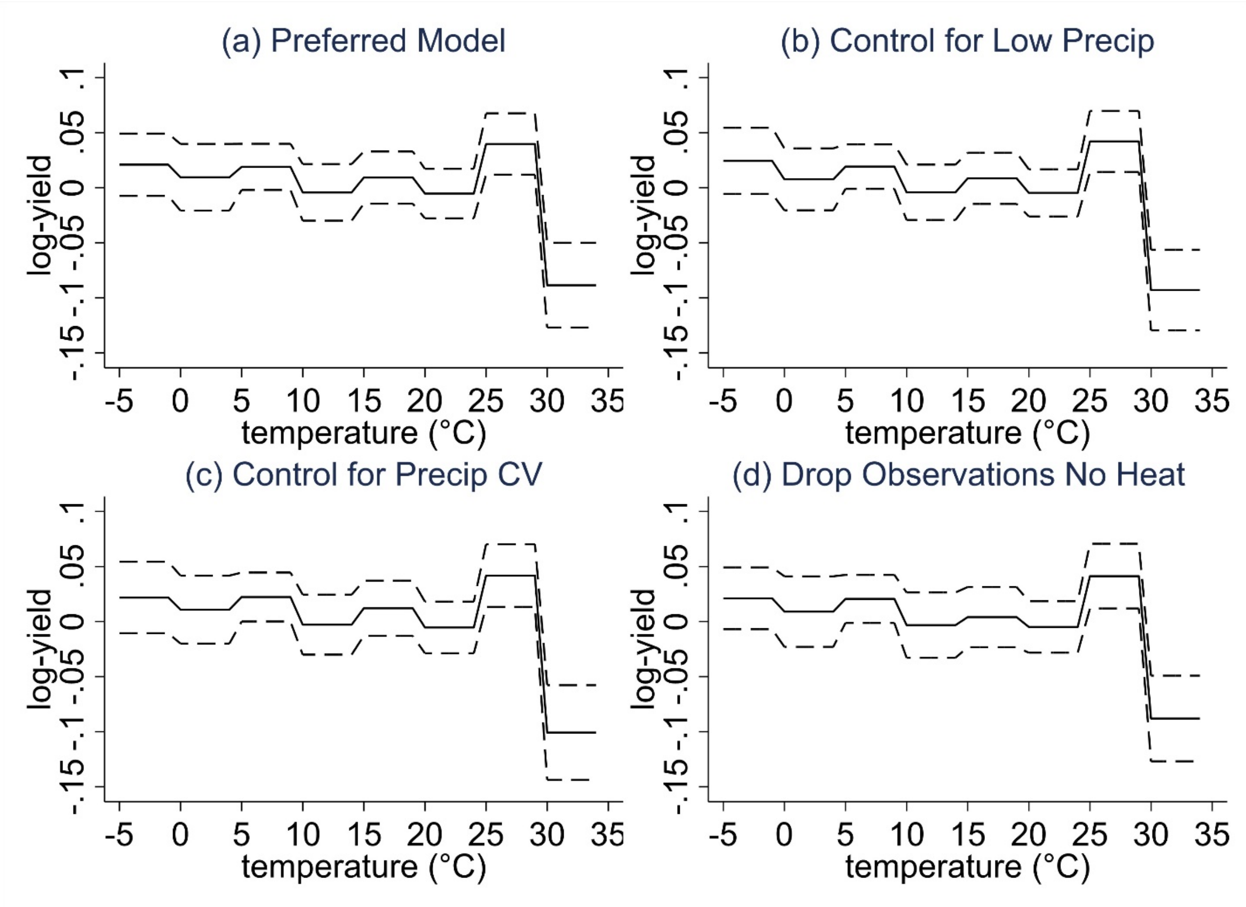

Supplementary Figure 9. Marginal Effects of Heat on Log Wheat Yield with Controls for Low Precipitation, Coefficient of Variation for Precipitation, and Dropping Observations without Heat Exposure. Panel (a) is the preferred model. Panel (b) represents the marginal heat effects controlling for an interaction with low precipitation (10th percentile). Panel (c) illustrates marginal heat effects while controlling for seasonal variation in precipitation via the precipitation coefficient of variation. Panel (d) shows the preferred model estimated only for site-year observations with heat exposure over 30°C. Dashed lines represent the 95% confidence interval with standard errors clustered by province-year.

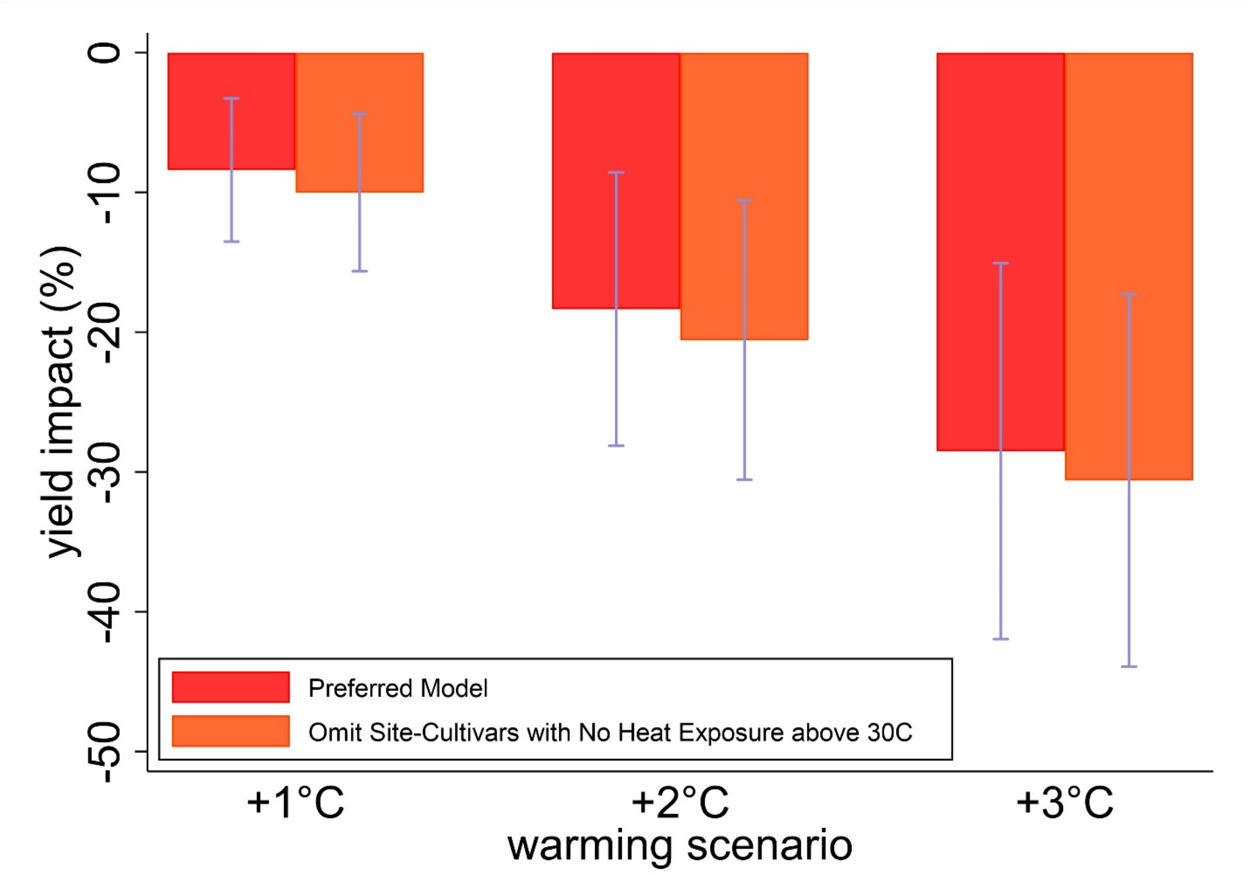

Supplementary Figure 10. Comparison of Warming Impacts with Restrictions on Site-Cultivar Heat Exposures. The preferred model includes all observations and the alternative model includes only cultivars that experienced heat exposures above 30°C at all locations in a given year. The preferred model is robust and captures similar heat exposures to those with restrictions on cultivar-locations included in the estimation. Bars show 95% confidence intervals using standard errors clustered by province-year for n=18,881 yield observations in the preferred model and n=17,306 yield observations omitting site-cultivars without heat exposure above 30°C.

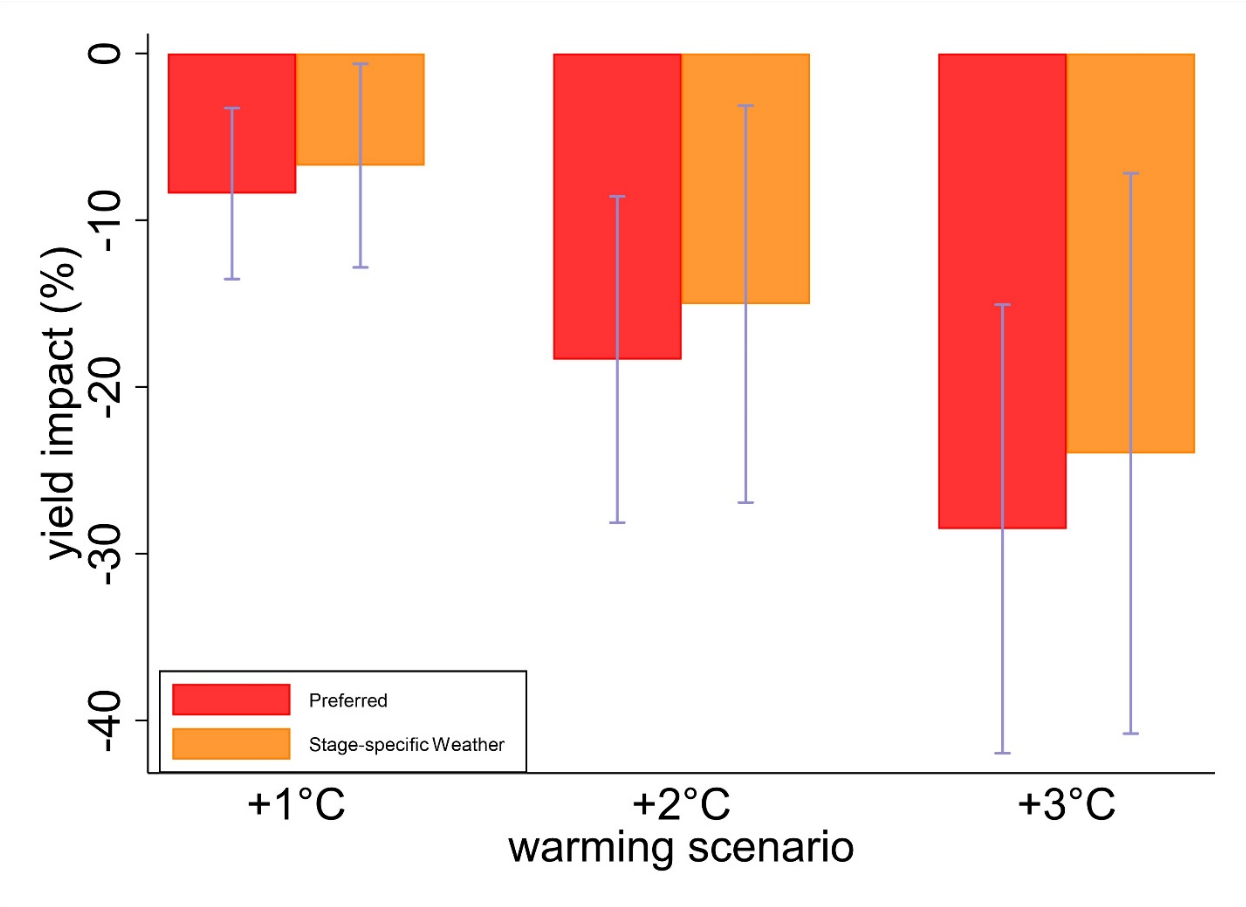

Supplementary Figure 11. Comparison of Warming Impacts in the Preferred Model with a Model allowing for Heat and Precipitation Effects to Vary by Growth Stage. Growing seasons were separated into three stages: (i) planting to 20 days before flowering to capture the vegetative stage, (ii) 20 days before to 10 days after the flowering date to capture the flowering stage, and (iii) 10 days after flowering to the end of season to capture the grain-filling stage. The alternative model was re-estimated including stage-specific measures of the precipitation and temperature variables. Findings suggest that warming impacts are very similar to those from our preferred model approach. Bars show 95% confidence intervals using standard errors clustered by province-year for n=18,881 yield observations.

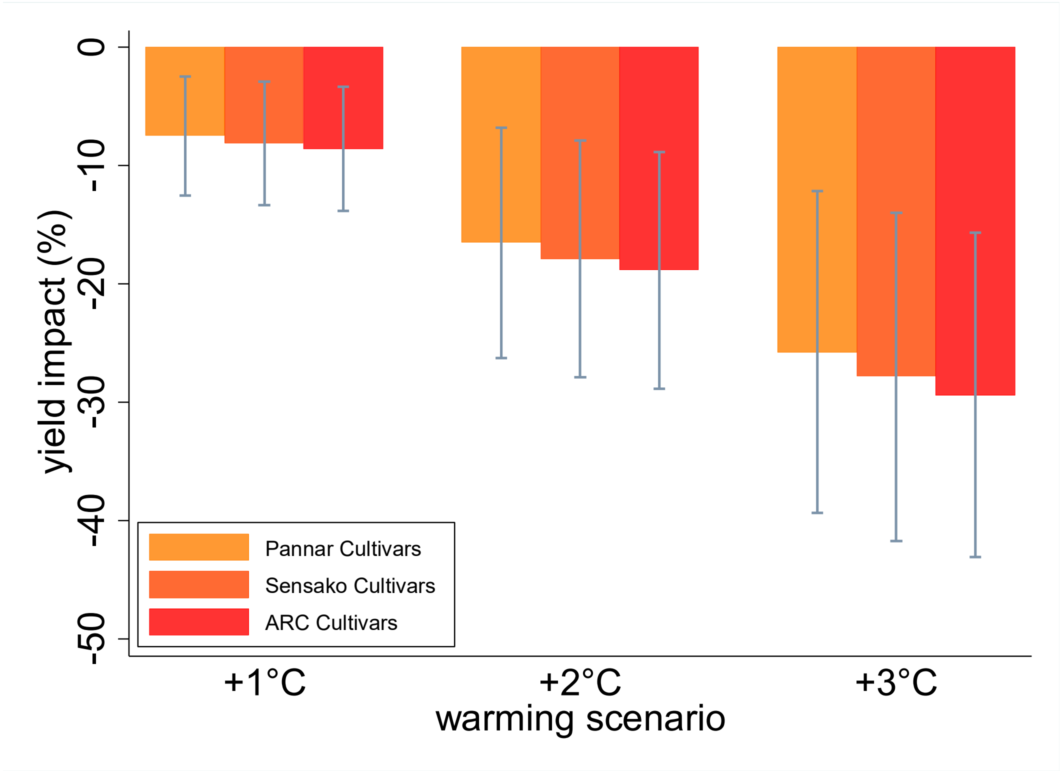

Supplementary Figure 12. Comparison of Warming Impacts on Wheat Yields Across Breeders. We allow the effect of temperature exposures above 30°C to vary across breeders. Impacts are reported as the percentage change in mean yield under +1 to +3°C warming scenarios relative to historical climate. Each 3-bar cluster shows warming impacts for the three largest South African wheat breeders: Pannar, Sensako, and ARC (Agricultural Research Council). Bars represent 95% confidence intervals using standard errors clustered by province-year for n=18,629 yield observations with breeder information. A two-sided joint test suggests statistically significant differences across breeders ($F(2,30) = 6.68$, p-value = 0.004), but the difference in yield effects is small.

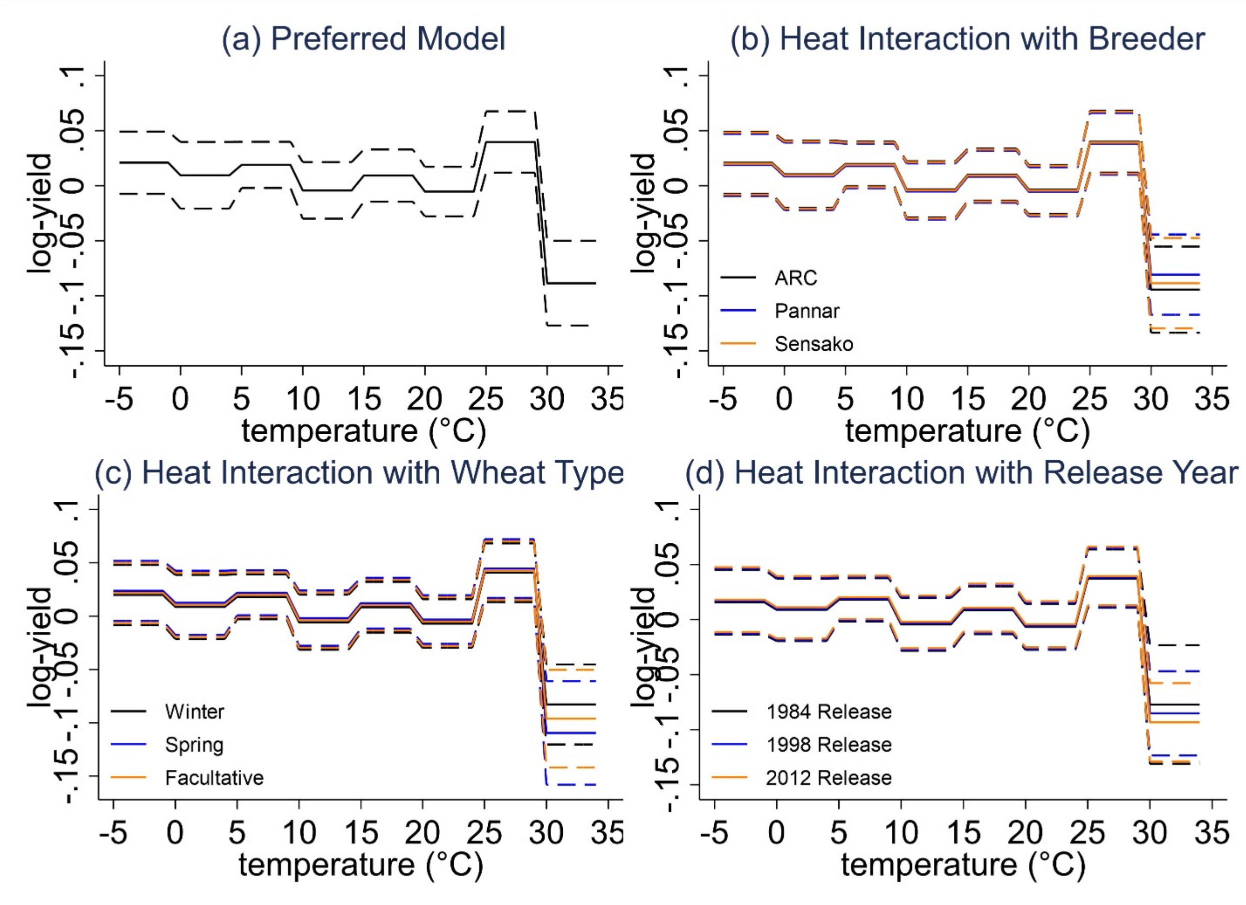

Supplementary Figure 13. Marginal Effects of Heat on Log Wheat Yield with Wheat Breeder, Wheat Type, and Release Year Interactions. Panel (b) shows the effects of ARC, Pannar, and Sensako breeder interactions, which are statistically different ($F(2,30) = 6.68$, p-value = 0.004) but at a very small magnitude. Panel (c) illustrates the interactions of heat with winter, spring, and facultative wheat, which were not statistically different ($F(2,30) = 2.20$, p-value = 0.128). Panel (d) presents interactions between heat and release year, which were not statistically different ($t(30) = 0.53$, p-value = 0.471). Dashed lines represent the 95% confidence interval with standard errors clustered by province-year.

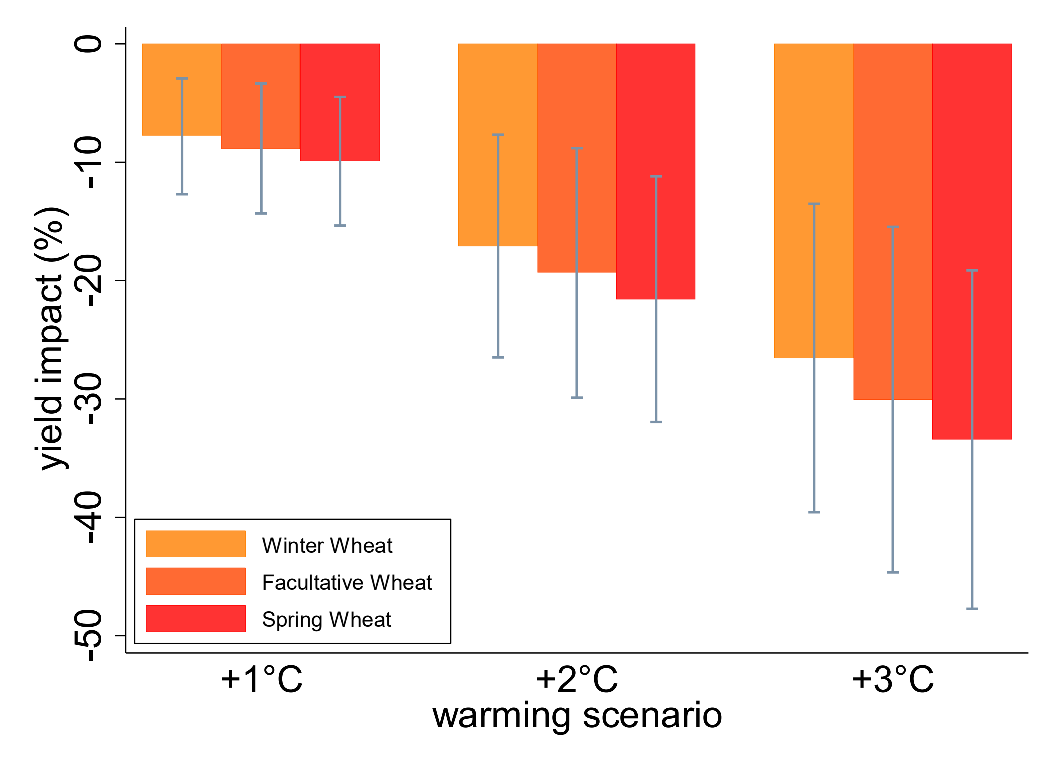

Supplementary Figure 14. Comparison of Warming Impacts on Yields Across Wheat Types. We allow the effect of temperature exposures above 30°C to vary across the three types of wheat represented in the data. Impacts are reported as the percentage change in mean yield under +1 to +3°C warming scenarios relative to historical climate. Each 3-bar cluster shows warming impacts for the three wheat types: winter, facultative, and spring. Bars show 95% confidence intervals using standard errors clustered by province-year for n=18,881 yield observations. A two-sided joint test suggests a lack of statistical significance for differences by wheat type ($F(2,30) = 2.20$, p-value = 0.128), and the warming impacts are similar across all three wheat types.

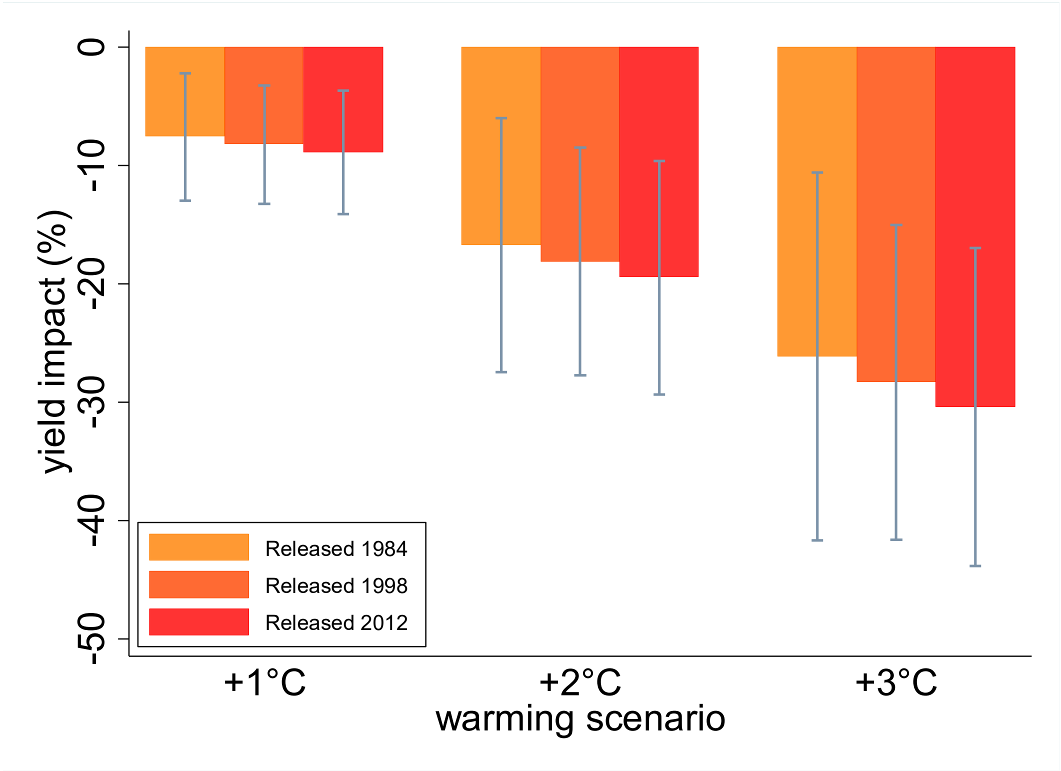

Supplementary Figure 15. Comparison of Warming Impacts on Wheat Yields across Time. We allow the effect of temperature exposures above 30°C to vary across time by including a linear trend interacted with the cultivar's release date. Impacts are reported as the percentage change in mean yield under +1 to +3°C warming scenarios relative to historical climate. Each 3-bar cluster shows mean warming impacts for the beginning, middle, and most recent release dates in the data: 1984, 1998, and 2012. Bars show 95% confidence intervals using standard errors clustered by province-year for n=18,230 yield observations with release year information. A two-sided test suggests a lack of statistical significance for the interaction (t(30) = 0.53, p-value = 0.471) coupled with similar warming impacts.

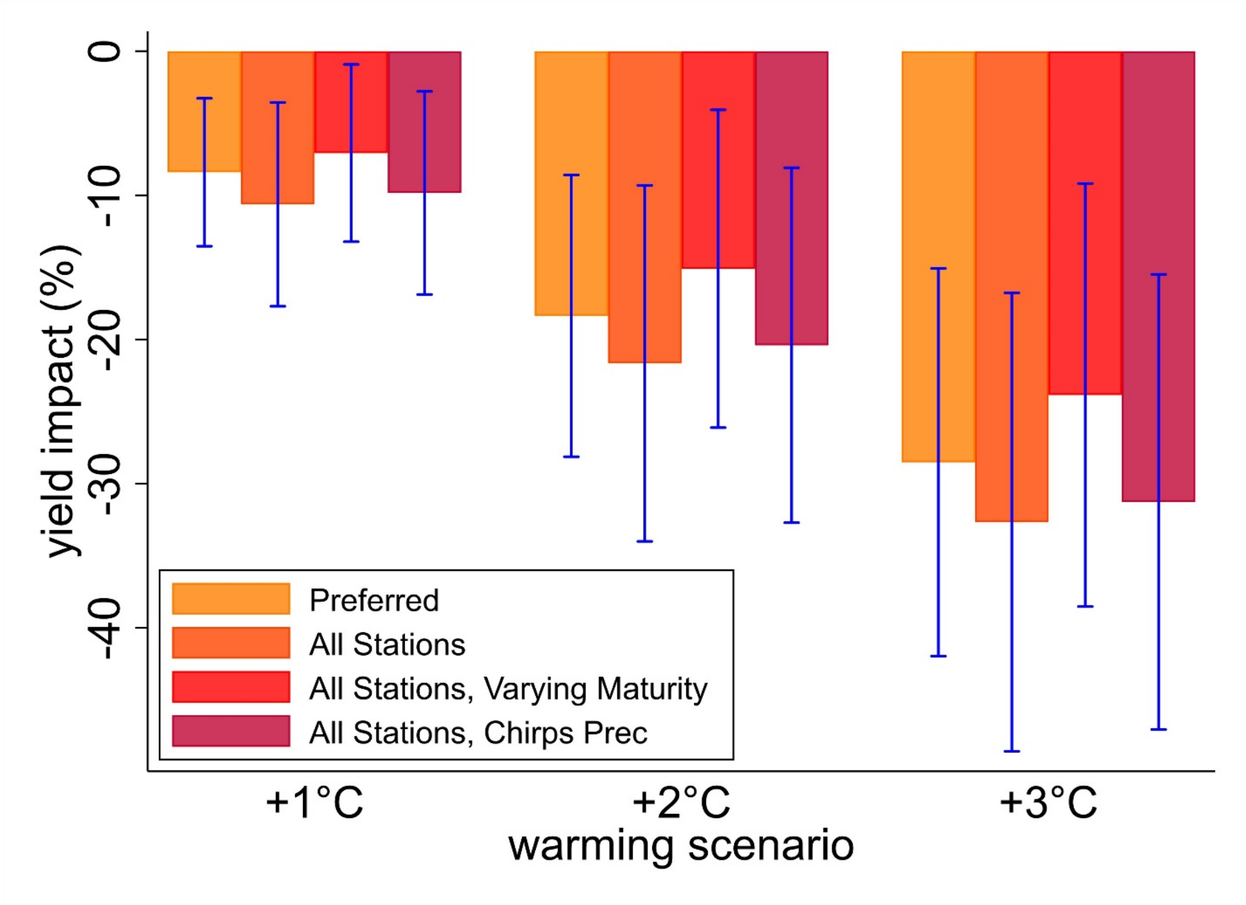

Supplementary Figure 16. Comparison of Warming Scenario Impacts on Wheat Yield with Daily Interpolations of Weather Station Data at Each Location, Different Maturity Periods, and CHIRPS Precipitation Data. The preferred model results with the original weather data and specification compared with weather interpolations. For every trial site, a distance-weighted (weight = $1/dist^2$) daily measure of tmin, tmax, and prec was interpolated using all stations within 200 km and then constructed the temperature exposure bins. In "all stations, fixed maturity", the maturity date was fixed at 30 days after flowering and the weather data was constructed from the interpolated daily data. The "all stations, varying maturity" represents interpolated weather data with optimal maturity period selection of 20 or 30 days by location-year. The warming impacts are similar (robust) to the preferred model in all alternative scenarios. Bars show 95% confidence intervals using standard errors clustered by province-year for n=18,881 yield observations in the preferred model and n=25,883 yield observations without restrictions on weather stations.

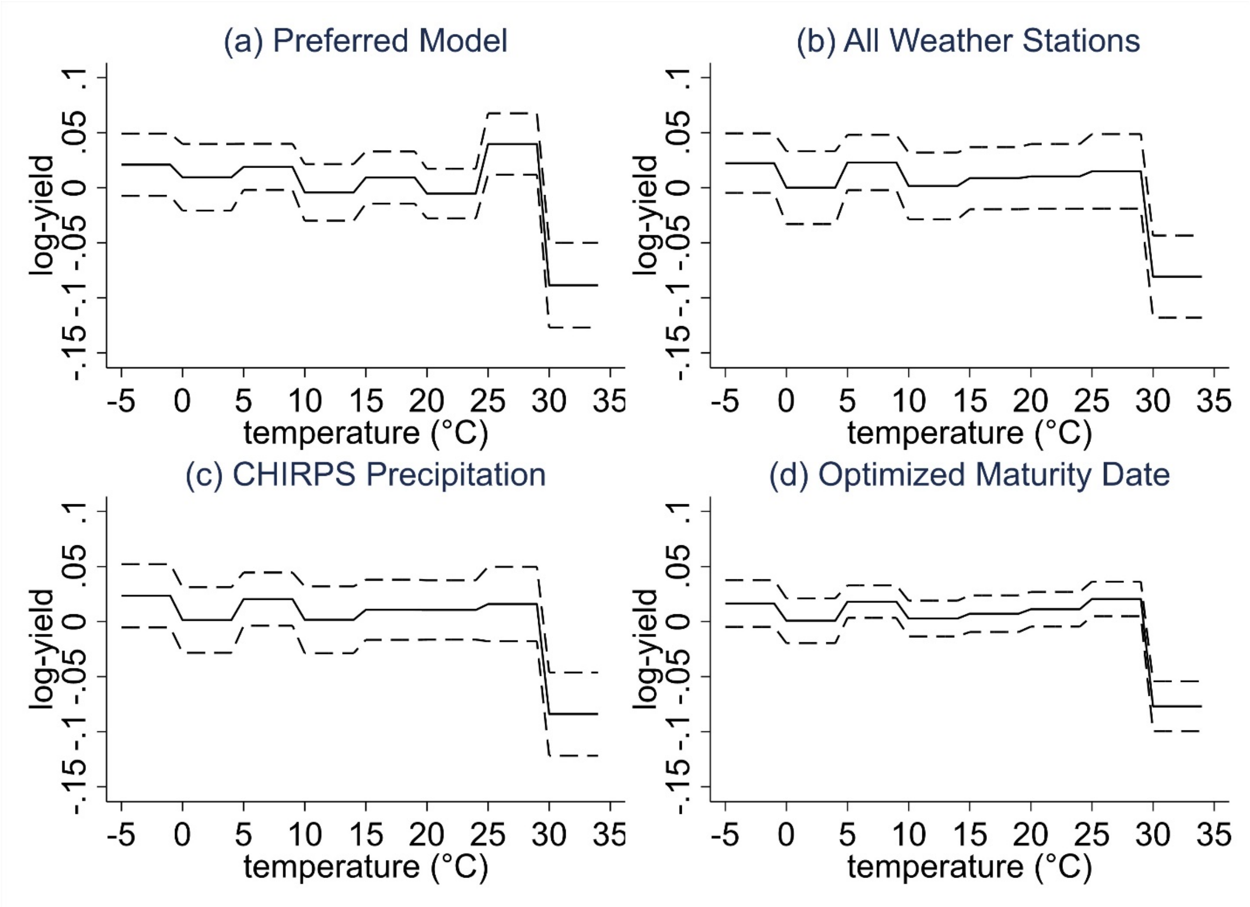

Supplementary Figure 17. Marginal Effects of Heat on Log Wheat Yield for the Daily Interpolations of Weather Station Data at Each Location, Different Maturity Periods, and CHIRPS Precipitation Data. The preferred model results with the original weather data and specification compared with weather interpolations. For every trial site, a distance-weighted (weight = $1/dist^2$) daily measure of tmin, tmax, and prec was interpolated using all stations within 200 km and then constructed the temperature exposure bins and the preferred model re-estimated. The CHIRPS gridded precipitation data was used to re-estimate the preferred model as a robustness check for precipitation. Finally, weather data was aggregated based on the optimal maturity period selection of 20 or 30 days by location-year. The warming impacts are similar (robust) to the preferred model in all alternative scenarios. Dashed lines represent the 95% confidence interval with standard errors clustered by province-year.

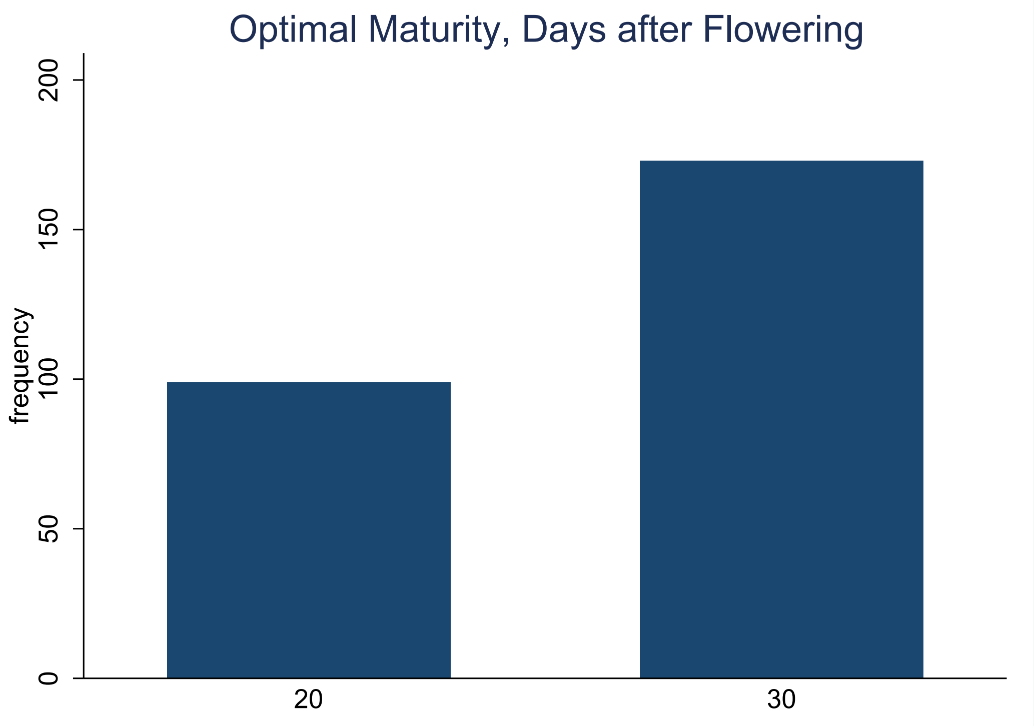

Supplementary Figure 18. Frequency of Optimal Maturity Period after Flowering for Weather Aggregation: 20 versus 30 days. The frequency of optimal maturity dates, as determined by r-squared values comparing models, suggests that 30 days from flowering to harvest accounts for more variation in most site-years.

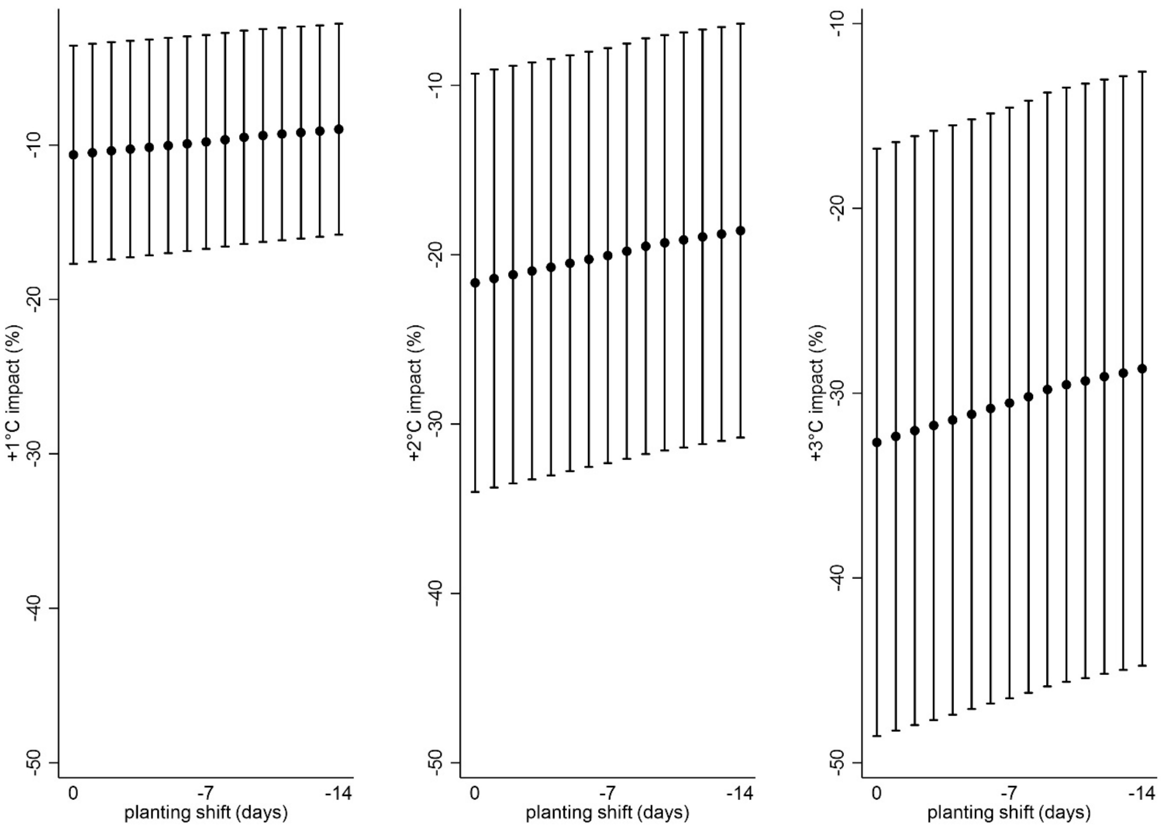

Supplementary Figure 19. Warming Impacts for One to Fourteen Days Earlier Planting Dates at +1-+3°C. The results suggest that planting 14 days earlier may reduce the impacts of +1°C warming by approximately 1 percent, and the earlier planting increasingly reduces impacts as temperatures warm up to +3°C at approximately 4 percent lower impacts compared to current planting dates. Bars show 95% confidence intervals using standard errors clustered by province-year for n=25,883 yield observations.

SUPPLEMENTARY TABLES

Supplementary Table 1. Characteristics of Sample Data across ARC-SGI Field Trial Sites

Site	# of Years	Minimum Year	Maximum Year	# of Cultivars	# of Yield Observations
Alpha	9	2005	2013	22	753
Arlington	7	2000	2012	40	901
Bethlehem	14	1998	2013	48	2655
Bultfontein	12	1998	2014	44	2707
Clarens	13	1998	2013	44	1466
Excelsior	5	1998	2007	33	597
Hopefield	10	1998	2013	26	629
Klipdale	7	2008	2014	14	184
Langgewens	12	1998	2012	25	1040
Malmesbury	6	2008	2014	17	300
Moorreesburg	12	1998	2014	25	579
Napier	7	2006	2014	22	355
Panorama	5	2008	2013	12	172
Philadelphia	10	1998	2012	25	411
Piketberg	10	1998	2013	25	456
Pools	7	2005	2013	22	444
Porterville	9	1998	2013	25	360
Protem	9	1998	2012	25	420
Reitz	10	1998	2009	40	1134
Riebeekwes	5	1998	2006	16	160
Senekal	6	1998	2009	35	808
Tygerhoek	8	2000	2011	26	936
Wesselsbron	10	1998	2012	44	1414
All Sites	17	1998	2014	71	18881

Note, while there are 23 specific ARC-SGI locations named, the sites represent only 17 locations in terms of latitude and longitude in the dataset. The following represent the same locations: (1) Arlington and Bethlehem; (2) Moorreesburg and Riebeekwes; (3) Alpha, Klipdale, Napier, Panorama, and Protem. ARC-SGI provide names of each of the cultivars tested for which a full list can be found in Supplementary Table 5.

Supplementary Table 2A. Mean Growing Season, Yield, Temperature, and Precipitation for Wheat Trial Sites

Site	Mean Plant Date	Mean Harvest Date	Yield (mt/ha)	Min T (°C)	Max T (°C)	Precipitation (mm)
Alpha	1-May	21-Sep	3.694	9.65	18.26	179.50
Arlington	13-May	6-Nov	1.711	2.20	21.12	125.59
Bethlehem	15-Jun	6-Dec	2.992	3.98	22.33	231.14
Bultfontein	11-May	4-Nov	3.717	6.26	24.33	63.25
Clarens	12-Jun	4-Dec	3.758	3.85	22.18	225.65
Excelsior	13-May	5-Nov	2.191	3.25	20.07	77.35
Hopefield	1-May	21-Sep	3.364	8.15	21.00	150.03
Klipdale	1-May	22-Sep	3.227	9.78	19.27	211.70
Langgewens	1-May	20-Sep	3.795	7.25	20.50	188.74
Malmesbury	1-May	22-Sep	4.303	6.99	20.70	204.54
Moorreesburg	1-May	21-Sep	4.274	7.27	20.83	228.09
Napier	1-May	21-Sep	3.483	9.65	18.52	197.00
Panorama	1-May	21-Sep	3.422	10.10	18.72	211.19
Philadelphia	1-May	21-Sep	4.545	7.07	20.60	186.95
Piketberg	1-May	20-Sep	3.759	7.22	20.59	208.06
Pools	1-May	21-Sep	3.585	7.43	20.57	250.46
Porterville	1-May	21-Sep	4.177	7.03	20.65	192.51
Protem	1-May	21-Sep	3.453	9.70	18.34	181.96
Reitz	10-Jun	2-Dec	2.344	3.69	22.19	202.96
Riebeekwes	1-May	19-Sep	3.960	7.55	21.33	172.85
Senekal	9-May	1-Nov	2.146	1.92	20.65	119.88
Tygerhoek	1-May	20-Sep	3.532	11.35	19.36	196.12
Wesselsbron	11-May	3-Nov	3.333	6.20	24.24	60.82
All Sites	17-May	26-Oct	3.314	5.97	21.63	163.78

Note, while there are 23 specific locations named, the sites represent only 17 locations in terms of latitude and longitude in the dataset. The following represent the same locations: (1) Arlington and Bethlehem; (2) Moorreesburg and Riebeekwes; (3) Alpha, Klipdale, Napier, Panorama, and Protem. The average seasons in Supplementary Tables 2B-D show the locational planting dates for winter, facultative, and spring wheats, respectively, while Supplementary Table 2A represents the average overall season weather for each location. Data for sites, planting date, and yield are from ARC-SGI. Harvest date was calculated based on the approach described in the methods. Weather data are derived from NASA GSOD.

Supplementary Table 2B. Mean Growing Season, Yield, Temperature, and Precipitation for Facultative Wheat Trial Sites

Site	Mean Plant Date	Mean Harvest Date	Yield (mt/ha)	Min T (°C)	Max T (°C)	Precipitation (mm)
Arlington	14-May	4-Nov	1.615645	2.180039	21.09282	123.823
Bethlehem	16-Jun	6-Dec	2.975637	3.996281	22.32766	233.6962
Bultfontein	12-May	3-Nov	3.649022	6.277664	24.247	64.62054
Clarens	12-Jun	3-Dec	3.641645	3.754618	22.10067	220.3646
Excelsior	14-May	3-Nov	2.166587	3.211	20.0346	77.13313
Reitz	11-Jun	2-Dec	2.282285	3.751845	22.21793	201.9643
Senekal	9-May	1-Nov	2.045217	1.899339	20.67858	114.845
Wesselsbron	11-May	2-Nov	3.168807	6.203326	24.15237	61.42375

Note, the following represent the same locations: (1) Arlington and Bethlehem; (2) Moorreesburg and Riebeekwes; (3) Alpha, Klipdale, Napier, Panorama, and Protem. The average seasons in Supplementary Tables 2B-D show the locational planting dates for winter, facultative, and spring wheats, respectively, while Supplementary Table 2A represents the average overall season weather for each location. Data for sites, planting date, and yield are from ARC-SGI. Harvest date was calculated based on the approach described in the methods. Weather data are derived from NASA GSOD.

Supplementary Table 2C. Mean Growing Season, Yield, Temperature, and Precipitation for Spring Wheat Trial Sites

Site	Mean Plant Date	Mean Harvest Date	Yield (mt/ha)	Min T (°C)	Max T (°C)	Precipitation (mm)
Alpha	1-May	21-Sep	3.694276	9.651045	18.2618	179.5042
Arlington	11-May	29-Oct	1.964	1.537539	20.39819	113.965
Bethlehem	14-Jun	15-Nov	3.924737	3.075876	21.37507	135.758
Bultfontein	11-May	29-Oct	3.509714	5.497541	24.18288	34.1954
Clarens	14-Jun	1-Dec	5.106563	4.037272	22.02712	236.8125
Hopefield	1-May	21-Sep	3.363672	8.154904	20.99917	150.0335
Klipdale	1-May	22-Sep	3.226957	9.784954	19.269	211.6987
Langgewens	1-May	20-Sep	3.795327	7.24566	20.5043	188.7394
Malmesbury	1-May	22-Sep	4.302933	6.992787	20.69632	204.5359
Moorreesburg	1-May	21-Sep	4.274007	7.270043	20.82959	228.0932
Napier	1-May	21-Sep	3.482592	9.653284	18.51944	196.9998
Panorama	1-May	21-Sep	3.422442	10.0994	18.72139	211.1871
Philadelphia	1-May	21-Sep	4.54472	7.074767	20.60229	186.9491
Piketberg	1-May	20-Sep	3.75932	7.219703	20.58636	208.059
Pools	1-May	21-Sep	3.584527	7.427674	20.56791	250.4649
Porterville	1-May	21-Sep	4.17675	7.029861	20.65273	192.5131
Protem	1-May	21-Sep	3.453429	9.701595	18.3375	181.9597
Reitz	1-Jun	30-Oct	4.103929	1.873863	21.20507	67.742
Riebeekwes	1-May	19-Sep	3.959812	7.5531	21.33335	172.8536
Tygerhoek	1-May	20-Sep	3.532382	11.35029	19.36225	196.1227
Wesselsbron	11-May	28-Oct	3.397949	5.339074	23.97617	35.40072

Note, the following represent the same locations: (1) Arlington and Bethlehem; (2) Moorreesburg and Riebeekwes; (3) Alpha, Klipdale, Napier, Panorama, and Protem. The average seasons in Supplementary Tables 2B-D show the locational planting dates for winter, facultative, and spring wheats, respectively, while Supplementary Table 2A represents the average overall season weather for each location. Data for sites, planting date, and yield are from ARC-SGI. Harvest date was calculated based on the approach described in the methods. Weather data are derived from NASA GSOD.

Supplementary Table 2D. Mean Growing Season, Yield, Temperature, and Precipitation for Winter Wheat Trial Sites

Site	Mean Plant Date	Mean Harvest Date	Yield (mt/ha)	Min T (°C)	Max T (°C)	Precipitation (mm)
Arlington	13-May	8-Nov	1.813	2.29	21.23	129.25
Bethlehem	12-Jun	9-Dec	2.930	4.06	22.47	234.78
Bultfontein	10-May	6-Nov	3.843	6.29	24.47	63.02
Clarens	12-Jun	8-Dec	3.918	4.04	22.37	236.44
Excelsior	12-May	8-Nov	2.248	3.35	20.16	77.87
Reitz	7-Jun	4-Dec	2.351	3.69	22.19	220.05
Senekal	7-May	4-Nov	2.436	1.97	20.58	134.40
Wesselsbron	10-May	6-Nov	3.613	6.25	24.40	61.75

Note, the following represent the same locations: (1) Arlington and Bethlehem; (2) Moorreesburg and Riebeekwes; (3) Alpha, Klipdale, Napier, Panorama, and Protem. The average seasons in Supplementary Tables 2B-D show the locational planting dates for winter, facultative, and spring wheats, respectively, while Supplementary Table 2A represents the average overall season weather for each location. Data for sites, planting date, and yield are from ARC-SGI. Harvest date was calculated based on the approach described in the methods. Weather data are derived from NASA GSOD.

Supplementary Table 3. Regression results for log wheat yield (mt/ha).

Variables	Preferred Model
Temp Exposure < 0C	-0.0151
	(0.0144)
Temp Exposure 0-4C	-0.0266*
	(0.0154)
Temp Exposure 5-9C	-0.0171
	(0.0107)
Temp Exposure 10-14C	-0.0403***
	(0.0131)
Temp Exposure 15-19C	-0.0268**
	(0.0121)
Temp Exposure 20-24C	-0.0413***
	(0.0115)
Temp Exposure 25-29C	0.00368
	(0.0142)
Temp Exposure > 30C	-0.125***
	(0.0196)
Precipitation	0.000750
	(0.00130)
Precipitation Squared	0.00000177
	(0.00000288)
R-squared	0.5552
Observations	18881
Locations	17
Cultivars	71
Years	17

Notes: Standard errors clustered by province-year are reported in parentheses. *, **, and *** denote statistical significance at the 10, 5, and 1 percent levels, respectively.

Supplementary Table 4. In and Out-of-Sample Comparison of Alternative Specifications.

Model	R-squared	Out of Sample RMSE (% reduction)
Preferred Specification	0.5552	-18.7
AS1: Linear time trend instead of year fixed effects	0.4845	-12.6
AS2: Quadratic time trend instead of year fixed effects	0.4847	-12.6
AS3: Add pre-season precipitation	0.5554	-18.7
AS4: Cubic polynomial for precipitation instead of quadratic	0.5556	-18.7
AS5: Quadratic avg daily temperature instead of exposures	0.5172	-15.4
AS6: Quadratic min/max daily temperature instead of exposures	0.5245	-16.0

Notes: RMSE is the percent reduction compared to a no-weather model with a linear trend.

Supplementary Table 5. Cultivar-level Heat Effects >30°C and Release Year.

Cultivar	Impact of >30°C	95% CI	Release Year
Baviaans	-0.1342606	[-0.2383268, -0.0389156]	2001
Bettadn	-0.1319167	[-0.186125, -0.0671819]	1993
Caledon	-0.1326621	[-0.1833614, -0.0700201]	1996
Elands	-0.1344276	[-0.1828839, -0.0778128]	1999
Gariep	-0.1262641	[-0.1782221, -0.0614911]	1994
Kariega	-0.1263351	[-0.2198382, -0.0134651]	1994
Komati	-0.1337049	[-0.1853121, -0.0738134]	2003
Limpopo	-0.1478313	[-0.2066959, -0.0822334]	1994
Matlabas	-0.1275661	[-0.1766092, -0.0713833]	2006
Pan3118	-0.1008664	[-0.1464812, -0.037422]	2003
Pan3120	-0.1115899	[-0.1646044, -0.0465639]	2005
Pan3144	-0.0982987	[-0.1365009, -0.044542]	2007
Pan3161	-0.1115895	[-0.1541562, -0.0394067]	
Pan3191	-0.118336	[-0.2655273, -0.0098663]	2002
Pan3235	-0.1240654	[-0.1778672, -0.0618598]	1995
Pan3349	-0.1089525	[-0.179153, -0.0393164]	1996
Pan3355	-0.1464203	[-0.2060698, -0.0655569]	2007
Pan3364	-0.1318196	[-0.1886594, -0.0740969]	1998
Pan3368	-0.1781459	[-0.2356032, -0.1208307]	2009
Pan3377	-0.1217599	[-0.1859154, -0.0598924]	1998
Pan3379	-0.127582	[-0.1763872, -0.0568687]	2010
Pan3408	-0.1276438	[-0.2028984, -0.0087943]	2003
Pan3434	-0.1221684	[-0.2019117, -0.0332101]	2006
Pan3471	-0.1180909	[-0.1670561, -0.0158456]	2012
Pan3492	-0.1288115	[-0.2207023, 0.0150168]	2002
Sst015	-0.1201383	[-0.1999399, 0.0156361]	2002
Sst027	-0.1405671	[-0.2631756, -0.0464368]	2004
Sst047	-0.1215784	[-0.2117001, -0.024085]	2006
Sst056	-0.126674	[-0.2386341, -0.0484893]	2006
Sst087	-0.1369773	[-0.3206386, -0.0710484]	2010
Sst124	-0.1055202	[-0.1746353, 0.0240504]	1987
Sst322	-0.1100032	[-0.2249228, -0.0084467]	2004
Sst333	-0.1368102	[-0.2313567, -0.0210214]	1993
Sst334	-0.1203893	[-0.2224353, -0.0377352]	2004
Sst347	-0.1191313	[-0.1643229, -0.0603084]	2006
Sst356	-0.1581631	[-0.212734, -0.0936464]	2006
Sst363	-0.1275334	[-0.2022008, 0.0029598]	1996
Sst367	-0.1592251	[-0.240276, -0.0040978]	1996
Sst374	-0.1390689	[-0.3159479, -0.0733938]	2010
Sst387	-0.1181876	[-0.1700817, -0.0408079]	2010
Sst399	-0.106449	[-0.161429, -0.0290246]	1999
Sst57	-0.132029	[-0.2285346, 0.0275906]	1996

Sst65	-0.1273089	[-0.196745, -0.0051102]	1997
Sst88	-0.1373372	[-0.2398395, -0.0398784]	1999
Sst966	-0.1168296	[-0.1746186, -0.0327198]	1996
Tankwa	-0.1341509	[-0.2500941, -0.0256587]	2009
Tugela-Dn	-0.1097353	[-0.158969, -0.0405442]	1992

Note, Supplementary Table 5 has 95% confidence intervals for the estimated heat impacts for each cultivar. These were constructed from block-bootstrapping whole years and re-estimating the model 10,000 times. Of the 47 cultivars, 5 (PAN3492, SST015, SST124, SST363, and SST57) were found to have a CI that contained 0, thereby suggesting a lack of statistical significance.
