# Supplementary material for: Yield reduction under climate warming varies among wheat cultivars in South Africa
Source: Nat Commun. 2020 Sep 2;11:4408. doi: 10.1038/s41467-020-18317-8 (PMC7468144; doi:10.1038/s41467-020-18317-8)
Supplement: Supplementary file 3 — Reporting Summary [file 41467_2020_18317_MOESM3_ESM.pdf]

nature research

Corresponding author(s): DBPR: NCOMMS-19-1125332B

Last updated by author(s): Aug 4, 2020

Reporting Summary

Nature Research wishes to improve the reproducibility of the work that we publish. This form provides structure for consistency and transparency in reporting. For further information on Nature Research policies, seeAuthors & Referees and theEditorial Policy Checklist .

Statistics

For all statistical analyses, confirm that the following items are present in the figure legend, table legend, main text, or Methods section.

n/a	Confirmed	
☐	☒	The exact sample size (*n*) for each experimental group/condition, given as a discrete number and unit of measurement
☐	☒	A statement on whether measurements were taken from distinct samples or whether the same sample was measured repeatedly
☐	☒	The statistical test(s) used AND whether they are one- or two-sided<br>*Only common tests should be described solely by name; describe more complex techniques in the Methods section.*
☐	☒	A description of all covariates tested
☐	☒	A description of any assumptions or corrections, such as tests of normality and adjustment for multiple comparisons
☐	☒	A full description of the statistical parameters including central tendency (e.g. means) or other basic estimates (e.g. regression coefficient) AND variation (e.g. standard deviation) or associated estimates of uncertainty (e.g. confidence intervals)
☐	☒	For null hypothesis testing, the test statistic (e.g. *F*, *t*, *r*) with confidence intervals, effect sizes, degrees of freedom and *P* value noted<br>*Give P values as exact values whenever suitable.*
☒	☐	For Bayesian analysis, information on the choice of priors and Markov chain Monte Carlo settings
☒	☐	For hierarchical and complex designs, identification of the appropriate level for tests and full reporting of outcomes
☒	☐	Estimates of effect sizes (e.g. Cohen's *d*, Pearson's *r*), indicating how they were calculated

Our web collection on statistics for biologists contains articles on many of the points above.

Software and code

Policy information about availability of computer code

Data collection	No software was used for data collection.
Data analysis	Stata version 15 was used to analyze data in this study. The codes used for statistical analysis in the current study are available in the wheatcultivars_heat_zaf repository on Github, https://github.com/amshew/wheatcultivars_heat_zaf.

For manuscripts utilizing custom algorithms or software that are central to the research but not yet described in published literature, software must be made available to editors/reviewers. We strongly encourage code deposition in a community repository (e.g. GitHub). See the Nature Research guidelines for submitting code & software for further information.

Data

Policy information about availability of data

All manuscripts must include a data availability statement. This statement should provide the following information, where applicable:

- Accession codes, unique identifiers, or web links for publicly available datasets
- A list of figures that have associated raw data
- A description of any restrictions on data availability

The datasets generated during and/or analyzed during the current study are available in the Harvard Dataverse repository, https://doi.org/10.7910/DVN/8Y6Q7F.

Field-specific reporting

Please select the one below that is the best fit for your research. If you are not sure, read the appropriate sections before making your selection.

☒ Life sciences ☐ Behavioural & social sciences ☐ Ecological, evolutionary & environmental sciences

Life sciences study design

All studies must disclose on these points even when the disclosure is negative.

Sample size	No sample size was chosen. The data used in this study were aggregated from the South African Agricultural Research Council's wheat trials. The wheat trials analyzed include 18,881 observations spanning 17 locations and 71 cultivars across 17 years, representing normal wheat production characteristics in South Africa.
Data exclusions	We only include wheat trial locations that have a weather station within 75 km and at least five years of wheat field trials, and wheat cultivars must appear in at least two trial years.
Replication	Table S1 summarizes the number of yield observations in the analysis by location, including the number of years the the location conducted trials, the first and last years of conducted trials, and the number of cultivars with replicated trials. Wheat trials were replicated in each location year with the same agronomic management and weather exposures. Yield observations by location year represent the average of the replicates in a given location-year. Wheat cultivars had to appear in at least two trial years and wheat trial locations had to be recorded in at least five years.
Randomization	All inputs, weather, and agronomic characteristics in wheat trials were the same for a given plot location and year, except where input treatments were recorded in the data (Table S1-S2D). Cultivars were tested in regression procedures while controlling for fixed effects by location and year. Standard errors were clustered by year-province to account for spatial autocorrelation. Location fixed effects control for all time-invariant factors at the location level (e.g. soil quality), while year fixed effects control for technological improvements over time that are common across locations. These include management improvements (e.g. increased fertilizer)and "stock" germplasm improvements that are common to all cultivars. Weather variables are included in the preferred regression model with temperature exposures in 5 degree C bins and a quadratic specification for precipitation (Table S3). The yield and weather data vary substantially in-sample, which supports robust estimation of wheat yield responses to extreme and average weather conditions (Supporting Tables S1, S2 and Figures S1, S2).
Blinding	This was not relevant for our study.

Reporting for specific materials, systems and methods

We require information from authors about some types of materials, experimental systems and methods used in many studies. Here, indicate whether each material, system or method listed is relevant to your study. If you are not sure if a list item applies to your research, read the appropriate section before selecting a response.

Materials & experimental systems

n/a	Involved in the study	
☒	☐	Antibodies
☒	☐	Eukaryotic cell lines
☒	☐	Palaeontology
☒	☐	Animals and other organisms
☒	☐	Human research participants
☒	☐	Clinical data

Methods

n/a	Involved in the study	
☒	☐	ChIP-seq
☒	☐	Flow cytometry
☒	☐	MRI-based neuroimaging
